# Supplementary material for: Associations between serum pigment epithelium-derived factor and physical performance in older women: The Otassha study
Source: PLoS One. 2025 Dec 16;20(12):e0338884. doi: 10.1371/journal.pone.0338884 (PMC12707620; doi:10.1371/journal.pone.0338884)
Supplement: S2 Table — (DOCX) [file pone.0338884.s002.docx]

**S2 Table. Relationship between Quartiles of Gait Speed and Skeletal Muscle Mass Index with Circulating lnPEDF Levels Adjusted for Potential Confounder (*n* = 143)**

|  | lnPEDF level | | | | |
| --- | --- | --- | --- | --- | --- |
| Quartile of Parameter | Q1 | Q2 | Q3 | Q4 |  |
|  | Mean (SD) | Mean (SD) | Mean (SD) | Mean (SD) | *p* |
| Gait Speed, n | 34 | 38 | 36 | 35 |  |
| Adjusted for age, Percent Body Fat, and DM | 2.444 (0.036) | 2.592* (0.034) | 2.566 (0.034) | 2.566 (0.035) | 0.019 |
| SMI, n | 34 | 39 | 38 | 32 |  |
| Age adjusted | 2.427 (0.037) | 2.629* (0.035) | 2.541 (0.035) | 2.567 (0.039) | 0.002 |

Values are least squares mean and SE adjusted for the factors by analysis of covariance. Q1–Q4: first to fourth quartile groups of gait speed or SMI, respectively. Data were analyzed by ANCOVA and Bonferroni post-hoc analysis. DM; diabetes mellitus. *p<0.05 vs. Q1.
